# Research Progress of Superhydrophobic Materials in the Field of Anti-/De-Icing and Their Preparation: A Review

**DOI:** 10.3390/ma16145151

**Published:** 2023-07-21

**Authors:** Qian Cong, Xiuzhang Qin, Tingkun Chen, Jingfu Jin, Chaozong Liu, Mingqing Wang

**Affiliations:** 1 Key Laboratory of Bionic Engineering (Ministry of Education), College of Biological and Agricultural Engineering, Jilin University, Changchun 130022, China; congqian@jlu.edu.cn (Q.C.); qinxz21@mails.jlu.edu.cn (X.Q.); jinjingfu@jlu.edu.cn (J.J.); 2 Department of Ortho and MSK Science, University College London, London HA7 4LP, UK; chaozong.liu@ucl.ac.uk; 3 Institute for Materials Discovery, University College London, London WC1E 7JE, UK; mingqing.wang@ucl.ac.uk

**Keywords:** superhydrophobic coating, anti-/de-icing methods, research progress, preparation methods, wear-resistant, self-healing/self-repairing, photothermal, electrothermal

## Abstract

Accumulated ice has brought much damage to engineering and people’s lives. The accumulation of ice can affect the flight safety of aircraft and lead to the failure of cables and power generation blades; it can even cause damage to human life. Traditional anti-icing and de-icing strategies have many disadvantages such as high energy consumption, low efficiency, or pollution of the environment. Therefore, inspired by animal communities, researchers have developed new passive anti-icing materials such as superhydrophobic material. In this paper, the solid surface wetting phenomenon and superhydrophobic anti-icing and de-icing mechanism were introduced. The methods of fabrication of superhydrophobic surfaces were summarized. The research progress of wear-resistant superhydrophobic coatings, self-healing/self-repairing superhydrophobic coatings, photothermal superhydrophobic coatings, and electrothermal superhydrophobic coatings in the field of anti-icing and de-icing was reviewed. The current problems and challenges were analyzed, and the development trend of superhydrophobic materials was also prospected in the field of anti-icing and de-icing. The practicality of current superhydrophobic materials should continue to be explored in depth.

## 1. Introduction

Ice adhesion is one kind of natural phenomenon that has many damages to engineering, such as aviation, transportation, and electric transmission. The adhesion of ice causes a reduction in lift, an increase in drag, and a reduction in the stall angle of the aircraft. Until 1988, 542 flight accidents have been caused by icing [1]. Ice adhesion can increase the weight of wind turbine blades and a reduction in the blade lift force, which can cause the motor to work poorly and reduce the system’s efficiency [2,3]. Ice accumulation during the rotation of the blades can fall off and may cause personal safety accidents. Even though some effective anti-icing and de-icing measures have been developed, they suffer from high energy consumption, high cost, and damage to material surfaces [4]. Therefore, it is important to study the corresponding materials for anti-icing and de-icing technology for daily life, industrial production, economy, and national construction.

Currently, researchers have conducted numerous studies to understand the underlying physicochemical mechanisms of icing and have developed many de-icing and anti-icing strategies [5,6]. Anti-icing and de-icing methods can be divided into active and passive methods. Active methods are through the use of external energy to de-icing, such as electrothermal de-icing, mechanical de-icing, chemical de-icing, etc. Electrothermal de-icing mainly applies current or voltage by the electric heating components so that the electrical energy is transformed into thermal energy and the heat energy can melt the ice [7]. M. Mohseni et al. [8] proposed the view of an embedded thermal element as an anti-icing and de-icing system for composite airfoils, in which an electric wire was buried into the composite airfoil as a thermal element, by which its surface could reach 20 °C and effectively prevent the surface from icing. However, this method has a large energy consumption and cost, and when the temperature is too high, it may cause damage to the surface material and even easily cause a fire. Mechanical de-icing is achieved through direct mechanical impact or vibration to break up the ice layer, which may way easily damage the road or material surface, and cable de-icing and other high-altitude operations are unsafe [2]. Chemical de-icing is mainly performed using substances to melt the ice, such as organic salts, glycols, and ethanol, but the frequent use of chemical methods for de-icing can have serious negative impacts on soil and water systems [9,10].

However, several of the above methods have defects such as complex design, high energy consumption, high cost, and environmental pollution, so it is important to research valid anti-icing methods. The passive methods refer to a chemical or physical modification of the surface so that the surface has the effect of rapid separation of water droplets from the substrate and delayed icing and can reduce the adhesion of ice to the substrate [11,12,13,14,15]. With the development of bionics, superhydrophobic surfaces were discovered by researchers and can be used for anti-icing. The surfaces with a contact angle of more than 150° and a rolling angle of less than 10° are defined as superhydrophobic surfaces [16,17,18,19,20]. Using a superhydrophobic surface to avoid ice formation is considered a promising method for anti-icing.

**Figure 1:**
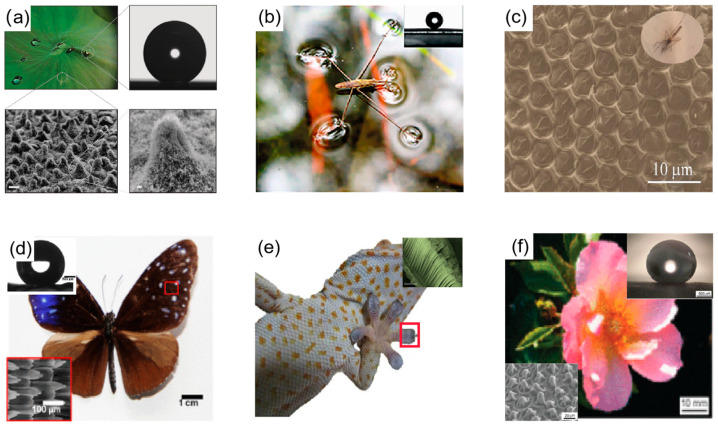
Animals and plants with superhydrophobic properties in nature. (**a**) The surface of a lotus leaf and its microstructure; optical image of a liquid drop on the surface of a lotus leaf [31]. (**b**) Six legs of a water strider standing on the water surface [26]. (**c**) SEM image of the microscale hemispherical eye of a mosquito [27]. (**d**) Optical view of a butterfly, with insets of the SEM image of the butterfly surface and the contact angle of the water droplet [28]. (**e**) Optical view of gecko with inset of SEM image of gecko foot [29]. (**f**) Scanning electron microscope image of a rose petal and an optical image of the contact angle of a water droplet on its surface [30].

In nature, a large number of plants and animals have superhydrophobic properties of their own. German botanists Barthlott and Neinhuis [21] revealed the surface microstructure of lotus leaves and suggested that the self-cleaning properties of lotus leaves is caused by micro/nano structures. The surface of the lotus leaf has micron-sized bumps and nanometer-sized wax crystals attached to the bumps, as shown in Figure 1a [22,23,24,25]. In addition to lotus leaves, there are other plants and animals with superhydrophobic properties in nature, such as the legs of water striders [26], eyes of mosquitoes [27], wings of butterflies [28], geckos [29], rose petals [30], etc., as shown in Figure 1. Inspired by the above-mentioned plants and animals, researchers have developed many superhydrophobic surfaces, but some superhydrophobic surfaces are not widely used due to their unstable properties and susceptibility to environmental and temperature influences. Therefore, this is the most important research task for the wide application of superhydrophobic surfaces.

This paper first introduces the solid surface wetting phenomenon and superhydrophobic materials’ anti-deicing mechanism and summarizes the preparation methods of superhydrophobic surfaces, then it reviews the research progress of wear-resistant superhydrophobic coating, self-healing/self-repairing superhydrophobic coating, photothermal superhydrophobic coating, and electrothermal superhydrophobic coatings in the field of anti-icing and de-icing in recent years. Finally, the current problems and challenges are analyzed. Based on this, it is pointed out that superhydrophobic materials are the focus and direction of future research in the field of anti-deicing, and the practicality of current superhydrophobic materials should continue to be explored in depth.

## 2. Solid Surface Wetting Phenomena and Anti-Deicing Mechanisms on Superhydrophobic Surfaces

### 2.1. Solid Surface Wetting Phenomena and Theory

#### 2.1.1. Young’s Equations

**Figure 2:**
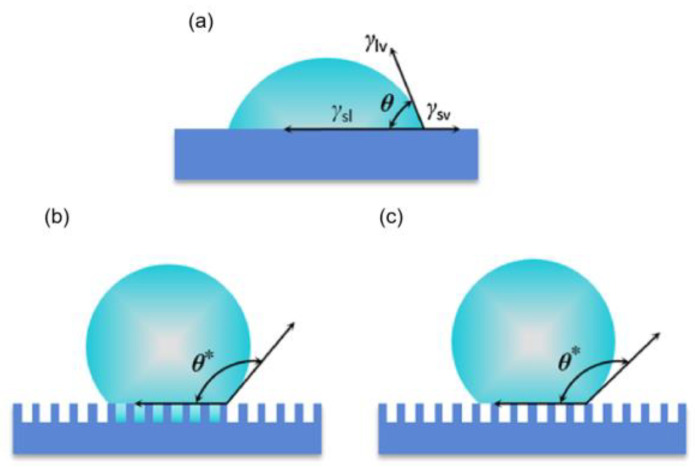
Schematic diagram of theoretical wetting models [35]. (**a**) Young’s equations; (**b**) Wenzel’s equation; (**c**) the Cassie–Baxter equation.

When a liquid drop falls on a solid, the liquid will be extended on the solid surface, and the original solid-gas interface and the liquid–gas interface will be transformed into a new solid–liquid interface; this phenomenon is defined as the wetting phenomenon. In 1805, Young [32], through continuous research on the phenomenon of wetting, proposed that when the three phases of solid, liquid, and gas are in balance on a smooth and homogeneous ideal surface, there is a certain relationship between the contact angle and the interfacial tension of the three phases of solid, liquid and gas, as shown in Figure 2a. The equation is as follows:

(1) $$\cos\theta =\frac{\gamma_{SL}-\gamma_{SV}}{\gamma_{LV}}$$

where *θ* is the contact angle; *γ_SL_* is the solid–gas phase interfacial tension; *γ_SV_* is the solid–liquid phase interfacial tension; and *γ_LV_* is the liquid–gas interfacial tension.

However, there are certain limitations of Young’s equation, which is only applicable to surfaces with uniform chemical composition and smoothness. In reality, most solid surfaces have an inhomogeneous chemical composition and certain roughness, so Young’s equation would no longer be applicable [2].

#### 2.1.2. Wenzel’s Equation

In 1936, Wenzel et al. [33] changed Young’s equation by introducing roughness. The Wenzel model assumes that a liquid can fill the grooves in the rough solid surface when liquid falls on the rough solid surface causing the liquid to be in complete contact with the solid, as in Figure 2b. The equation is as follows:

(2) $$\cos\theta^{*}=r\cos\theta$$

where θ* is the apparent contact angle of the rough surface and r is the roughness factor (the ratio of the actual contact area of the solid–liquid to the apparent contact area of the solid–liquid).

When r = 1, the solid surface is ideally smooth and the Wenzel equation is the same as Young’s. When r > 1, for hydrophobic surfaces, θ* will increase with the increase of roughness r, and for hydrophilic surfaces, θ* will decrease with the increase of roughness r. This further indicates that in Wenzel’s equation, increasing the solid surface roughness makes hydrophilic surfaces more hydrophilic and hydrophobic surfaces more hydrophobic. However, the equation also has some limitations; the equation does not apply when the chemical composition of the solid surface is different. Many other wetting phenomena in nature cannot be explained by the Wenzel equation, such as the lotus effect.

#### 2.1.3. The Cassie–Baxter Equation

Due to the limitations of Wenzel’s equation, Cassie and Baxter [34] further extended Wenzel’s equation by suggesting that when a liquid falls on a rough solid surface, the air in the grooves of the rough surface is trapped by the liquid, forming both a solid–liquid interface and a gas–liquid interface at the interface, as shown in Figure 2c. At this point, the contact angle of the liquid will have two components, namely, the contact area of the liquid with the solid surface and the contact area of the liquid with the air trapped in the groove. The apparent contact angle θ* of a solid surface can be expressed by Cassie–Baxter as follows:

(3) $$\cos\theta^{*}=f_{1}\cos\theta_{1}+f_{2}\cos\theta_{2}$$

where *f*_1_ is the area fraction of solid–liquid contact; *f*_2_ is the area fraction of gas–liquid contact, where *f*_1_ + *f*_2_ = 1. *θ*_1_ represents the contact angle of solid–liquid; *θ*_2_ represents the contact angle of liquid–gas. As the contact angle between water and air *θ*_2_ = 180°, the above equation can be rewritten as follows:

(4) $$\cos\theta^{*}=f_{1}\cos\theta_{1}+f_{1}-1$$

The Cassie–Baxter equation assumes that the liquid droplet is only partially in contact with the solid surface and the rest of the droplet is in contact with air. This equation illustrates that the microstructure of a superhydrophobic material can effectively keep air underneath the droplet so that the liquid droplet cannot enter the micro-nanostructure. The theory is of great importance for future research on superhydrophobic materials.

### 2.2. Superhydrophobic Anti-/De-Icing Mechanism

Since researchers discovered the superhydrophobic characteristics of plants and animals such as lotus leaves [36] and mosquito eyes [37], many researchers have started to imitate the characteristics of plants and animals with superhydrophobic properties to prepare superhydrophobic materials with micro/nano structures. Currently, there are three main principles for preventing ice formation on superhydrophobic surfaces: (1) reducing the residence time of water droplets on superhydrophobic surfaces (causing water droplets to roll off the surface before they nucleate and freeze) [38,39,40]; (2) delaying the freezing time of water droplets [41,42]; and (3) reducing the adhesion of ice even if water droplets freeze on superhydrophobic surfaces [43,44].

#### 2.2.1. Reducing the Residence Time of Water Droplets on Superhydrophobic Surfaces

**Figure 3:**
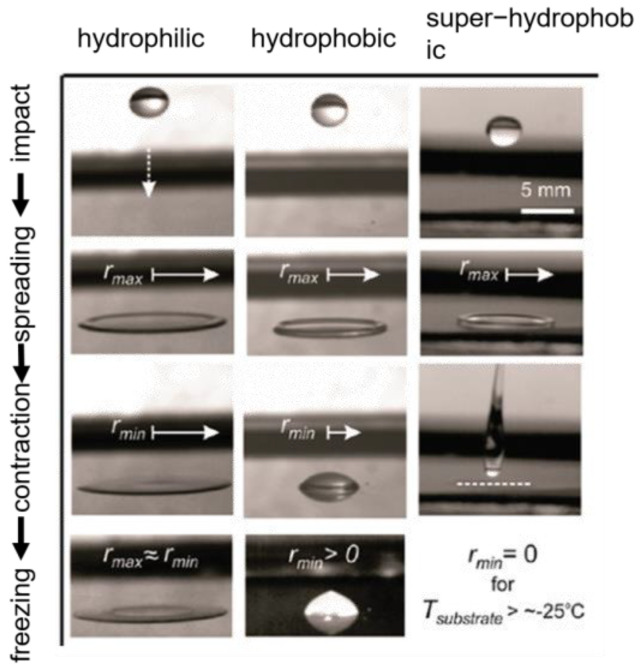
Continuous dynamic action of a 15 µL water droplet impacting different cooling surfaces from a height of 10 cm [51].

Surfaces with a contact angle of more than 150° and a rolling angle of less than 10° are generally defined as superhydrophobic surfaces. Superhydrophobic surfaces usually have micro/nano structures. When a water droplet falls on a superhydrophobic surface, the air is trapped in the micro-nanostructure, forming a Cassie–Baxter model of solid–liquid–gas three-phase contact [45,46] and reducing the interaction between the droplet and the surface, and when the surface is tilted, the droplet slides on the superhydrophobic surface [40,47,48,49,50]. In addition, due to the low contact angle hysteresis (CAH < 5°) of superhydrophobic surfaces, droplet bounce behavior will occur when a water droplet comes into contact with a superhydrophobic surface at an initial velocity; the droplet will first spread into a pancake shape. This process can store kinetic energy; the stored energy then causes the droplet to shrink into a sphere and finally rebound from the superhydrophobic surface [51]. As shown in Figure 3, droplets adhere to and freeze on hydrophilic and hydrophobic surfaces because they do not fully retract when they impact, whereas impacting droplets on superhydrophobic surfaces can fully recoil and rebound before freezing occurs. In summary, superhydrophobic surfaces can remove water droplets before they freeze [41].

#### 2.2.2. Delaying the Freezing Time of Water Droplets

In nature, ice formation is a widespread phenomenon. When the temperature falls below 0 °C, water droplets will undergo nucleation over a period of time [52]. Water droplet freezing is the process of nucleation of supercooled water. Ice nucleation is divided into homogeneous nucleation and non-homogeneous nucleation. Under ideal circumstances, the process of ice nucleation is not affected by other impurities or external surfaces and the probability of forming a critical nucleus is accordant throughout the system, which is called homogeneous nucleation. Homogeneous nucleation usually occurs at around −40 °C. Homogeneous nucleation rarely occurs in most ice formation processes. When the ice nucleation process is influenced by impurities or external surfaces, nucleation will be promoted, and extensive nucleation occurs on the impurities or external surfaces, which is called non-homogeneous nucleation. Non-homogeneous nucleation normally happens when the water is chilled to roughly 0 °C. Therefore, delaying the freezing period entails increasing the nucleation time of the ice. The generation of the nucleation process requires overcoming the free energy nucleation barrier [53]. The homogeneous phase nucleation Gibbs free energy barrier is as follows:

(5) $$\Delta F_{homo}=\frac{16\pi \gamma^{3}}{3{\left(\rho_{n}\Delta \mu \right)}^{2}}$$

where *ρ*_n_ is the number density of nucleating phase; Δ*μ* is obtained by subtracting the chemical potential of the nucleating phase (water) from the chemical potential of the forming phase (ice); and γ is the tension at the ice—water interface. Homogeneous nucleation, which is described by the above equation, can only occur if the ice nucleus reaches the critical size R*:

(6) $$R^{*}=\frac{2\gamma }{\rho_{n}\Delta \mu }$$

In non-homogeneous nucleation, the nucleation barrier can be expressed as follows:

(7) $$\Delta F_{HETERO}=\Delta F_{HOMO}f\left(m,x\right)$$

(8) $$x=\frac{R}{R^{*}}$$

where *m* is the contact angle formed between the nucleated phase body (liquid water when ice is crystallized) and the solid surface at the contact interface; *x* is the ratio of surface roughness radius of curvature (*R*) to the critical radius (R*).

From Equation (7), it is clear that the ability to delay ice nucleation is mainly determined by the wettability and surface roughness of the solid surface. Superhydrophobic surfaces have lower surface energy and larger roughness, which can delay the time of ice nucleation. In addition, water droplets on superhydrophobic surfaces have a large contact angle. The higher the contact angle of water droplets on the superhydrophobic surface, the smaller the solid–liquid contact area and the larger the liquid–gas contact area; an air layer with low thermal conductivity is formed, thereby weakening the heat transfer from the hotter water droplets to the colder superhydrophobic surface and reducing the possibility of non-homogeneous nucleation at the water–superhydrophobic interface [54,55,56].

#### 2.2.3. Reducing the Adhesion of Ice

The adhesion force of the ice is closely connected to the real contact area between the ice and the solid surface. Superhydrophobic surfaces may reduce the adhesion force of ice, according to Sarkar et al. [57]. As the actual contact area shrinks, so does the adhesion force of ice. Even if water droplets freeze on a superhydrophobic surface, the ice adhesion force is reduced due to the minimal actual contact between the ice and the surface, and the ice slips off naturally when external forces are applied (wind, gravity, etc.).

Additionally, the strength of ice adherence is correlated with the surface’s wetting hysteresis. According to studies comparing the adhesion of a superhydrophobic surface with that of a normal surface, a superhydrophobic surface with low hysteresis has lower ice adhesion [43]. Huang et al. [58] tested the ice adhesion of superhydrophobic coatings, room temperature vulcanized silicone rubber (RTV) coatings, and bare glass panels and concluded that the ice adhesion of superhydrophobic coatings was much lower than that of RTV coatings and bare glass panels. This is also due to the micro and nanostructure of the superhydrophobic surface, which allows for a smaller contact area between the ice and the surface. However, not all superhydrophobic surfaces can reduce ice adhesion, and it is controversial that superhydrophobic surfaces can reduce the adhesion of ice [59,60].

## 3. Methods for the Preparation of Superhydrophobic Surfaces

A surface’s superhydrophobic properties is primarily influenced by surface free energy and surface roughness [30,61,62,63,64]. A surface’s superhydrophobic properties rises with rising surface roughness and with falling surface free energy. Therefore, altering the surface roughness and lowering the substance’s surface free energy are the two key steps in creating superhydrophobic surfaces.

### 3.1. Template Method

A surface with low surface energy is used as a template in the template method. Materials such as polymers are then extruded or poured to mimic the rough structure on the template and then demolded to produce a superhydrophobic surface. Soft and hard templates make up the two categories of frequent sorts of templates. Polymer templates such as Polytetrafluoroethylene (PTFE), polypropylene, and polydimethylsiloxane are the most common types of soft stencils. Metal and glass stencils are the two most common types of hard templates.

**Figure 4:**
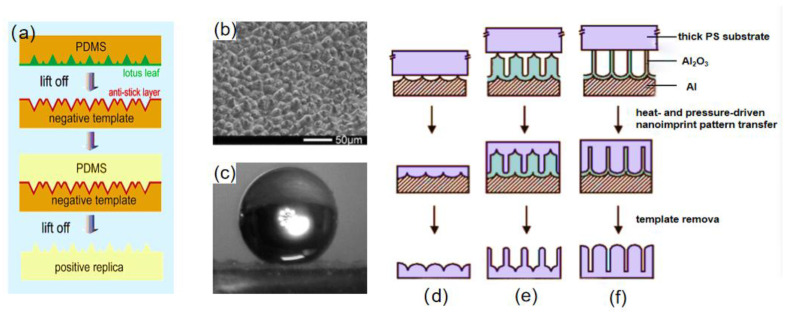
(**a**) Replication process of a PDMS superhydrophobic surface [65]. (**b**) SEM image of a PDMS superhydrophobic surface [65]. (**c**) Image of a water droplet on a PDMS superhydrophobic surface [65]. Process diagram for fabrication of nanoimprint patterns driven by heat and pressure for nanofabrication of the surface of the thick polymer substrate with (**d**) aligned nanoemboss, (**e**) nanopost array with embossed base, and (**f**) aligned nanofibers [66].

The natural substrate may be duplicated using the template method, and the superhydrophobic structure of the plants and animals can be accurately modeled. Sun et al. [65] used the two-template method to create the superhydrophobic surface. The mixture of Polydimethylsiloxane (PDMS) and catalyst was poured onto the surface of the lotus leaf, after curing at room temperature, and the PDMS was stripped from the leaf to obtain a soft stencil with a complementary microstructure to the lotus leaf. The previous step was repeated; the mixture of PDMS and catalyst was poured onto the soft stencil, and after curing, the PDMS was stripped to obtain a superhydrophobic surface, as shown in Figure 4a. As seen in Figure 4b,c, the surface had a microstructure resembling that of a lotus leaf, and water droplets were spherically on it.

Liu et al. [67] poured PDMS prepolymer onto fresh lotus leaves; the PDMS prepolymer was cured at 40 °C for 4 h and demolded to obtain a soft PDMS template. The epoxy-based azo polymer was dissolved in tetrahydrofuran (solution concentration of 35 mg·mL^−1^), and the solution was dripped onto the surface of the substrate. The prepared PDMS template was pressed onto the surface of the substrate, and after some time, the PDMS template was peeled off and the surface of the substrate was placed in a vacuum oven at 25 °C for 6 h to obtain a superhydrophobic surface. The surface’s roll angle was less than 10°, and its contact angle was larger than 150°.

Nanoimprint lithography is also a technique that can replicate surfaces features, which is mainly done by heat and pressure and is capable of replicating morphologies of nanometers [68]. Lee et al. [66] prepared nano-polystyrene (PS) surfaces with controllable stretch ratios by using nanoimprinting, as shown in Figure 4d–f. Firstly, textured aluminum sheets and nanoporous alumina were prepared as replica templates, then a certain amount of heat and pressure was applied to drive the polymer melt to impregnate the nanopatterns of the replication templates, and, finally, the replication template was removed from the polymer substrate by reacting with Al in saturated HgCl_2_ solution to obtain a large area of nanostructured PS surface. The contact angle of the synthesized surface was between 155.8° and 147.6° and the synthesized surface was slightly tilted; water droplets that fall on the superhydrophobic surface will roll out. It is worth paying attention to the circumstance that the thickness of the replica template can be varied to control the length of the PS nanofibers.

**Figure 5:**
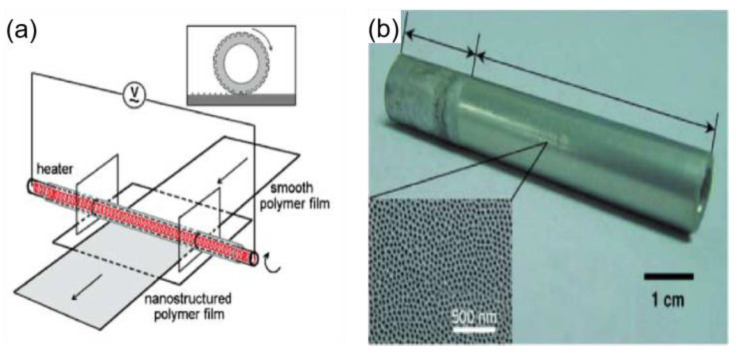
(**a**) Rolling on a film using a tubular template at the right temperature and pressure [69]. (**b**) Tubular porous anodized aluminum template; inset is an SEM image of the surface [69].

Guo et al. [69] used a similar method to prepare a superhydrophobic surface, the porous anodic aluminum oxide was used as a template, and the porous anodic aluminum oxide was rolled on a polycarbonate (PC) under appropriate pressure and temperature conditions, which formed a well-aligned polycarbonate (PC) nanopore array surface. This method modifies the originally hydrophilic PC surface to a hydrophobic PC surface and can be used for large-area preparation, as shown in Figure 5.

Alternatively, a manual template can also be obtained by etching the substrate. In order to create hard templates, Wu et al. [70] etched aluminum alloy through hydrochloric acid (2 mol/L), and then immersed it for 30 min in an ethanol solution containing N-dodecyltrimethoxysilane (0.01 mol/L). In order to create a PDMS superhydrophobic surface, Sylgard 184 Silicone was mixed in a 10 to 1 weight ratio of elastomer and curing agent and then poured into the hard templates. After being cured at 60 °C for two hours, the PDMS was stripped from the hard templates. This superhydrophobic surface offered a 165° contact angle, good self-cleaning capabilities, and good water droplet bounce.

The template method is simple, reproducible, low cost, and can quickly replicate the rough structure of the stencil surface.

### 3.2. Coating Method

#### 3.2.1. Solution Spraying Method

To provide the substrate’s superhydrophobic properties, the particles with low surface energy can be sprayed on the substrate’s surface. The method allows any material to be selected as the substrate, such as metal, glass, fabric, etc. It can be prepared across a sizable area, and if a portion of the coating fails, the solution can be resprayed to the failed area.

**Figure 6:**
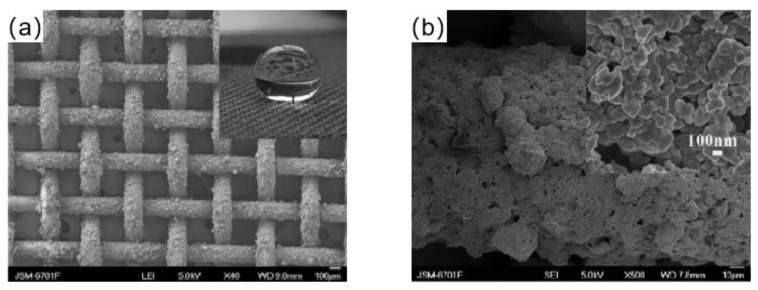
(**a**) SEM images of the copper mesh after coating [71]. (**b**) FESEM images of copper mesh wire after spraying [71].

Xu et al. [71] prepared a superhydrophobic copper mesh by spraying method. An amount of 0.42 g of n-octadecanethiol was mixed with 20 mL of ethanol and swirled for 20 min at room temperature to create a homogenous solution. Then, the homogeneous solution was mixed with 20 mL of an ethanol solution of silver nitrate and agitated for 60 min. Using the spraying technique, the reaction solution was applied to the copper mesh. When C_18_H_37_SAg was produced on the copper mesh surface and its alkyl end was exposed to the air, the free energy on the copper mesh surface was reduced, creating a superhydrophobic surface, as shown in Figure 6.

Similarly, Elszaabalawy et al. [72] used a spraying method to prepare a superhydrophobic coating with multifunctional properties. The two-component silicone polymer was dissolved in 40 mL of acetone solvent and sonicated for 10 min. Then, 1 g of functionalized SiO_2_ nanoparticles were added to the dispersions and the mixed solution was sprayed onto the substrate using a spray gun at the prescribed air pressure. The coated substrates were cured at 120 °C for an hour and then cured at room temperature for 24 h to obtain a superhydrophobic coating. The coating provided superhydrophobic properties when the SiO_2_ concentrations were greater than 9 wt%, and the coating maintained superhydrophobic properties under hot and corrosive conditions. However, the coating created with this technique generally has poor adherence strength at the substrate-to-coating interface.

Therefore, Rafik Abbas et al. [73] proposed a method to improve the adhesion between substrate and coating by adding a mucoadhesive polymer. The functionalized silica nanoparticles modified with octyltriethoxysilane (FS) were mixed with epoxy resin (Epoxy) and sprayed on different substrate surfaces with a spray gun, and then the substrate was cured to obtain a superhydrophobic coating with micro and nano-graded structure. The adhesion of the coatings was tested with tape, and the adhesion of the coatings increased with the content of the epoxy resin, as shown in Table 1. This further demonstrates that the use of adhesives does increase the coating’s adherence to the substrate.

#### 3.2.2. Chemical Deposition Method

Chemical deposition is a process whereby the substrate reacts with a chemical solution to form a coating with a rough structure on the substrate. For example, Zhang et al. [74] prepared a superhydrophobic coating with a layered structure and bimetallic composition by chemically depositing Ag onto a copper surface. In addition, the superhydrophobic coating had the added benefit of enhanced antibacterial activity. Chen et al. [75] prepared a superhydrophobic gold–zinc alloy surface by chemical deposition and annealing treatment. The prepared superhydrophobic surface had a contact angle of up to 170° and a roll angle of less than 1°, providing a significant superhydrophobic effect without any modifier incorporation.

The chemical deposition method is low-cost, simple, and reproducible, and the superhydrophobic coatings obtained by this method have good chemical stability. However, it also has certain drawbacks, and the addition of chemical substances can cause some damage to the human body and the environment.

In addition, according to the difference in deposition conditions, electrochemical deposition and chemical vapor deposition are proposed.

#### 3.2.3. Electrochemical Deposition Method

**Figure 7:**
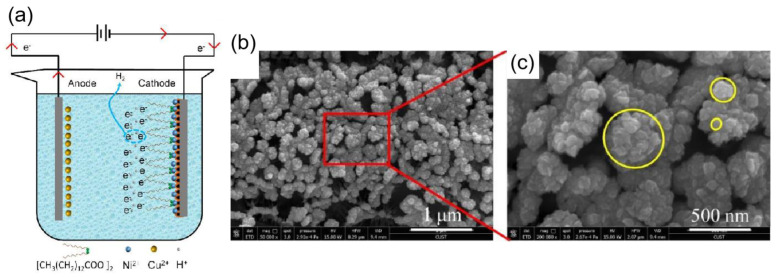
(**a**) Schematic diagram of the electrodeposition process to prepare a superhydrophobic nickel film [76]. (**b**) SEM image of the nickel film after 10 min of electrolysis [76]. (**c**) Enlarged SEM image of the red line part [76].

Electrochemical deposition is a technique for crystallizing metals or oxides by causing the cations and anions in a solution to migrate and produce redox reactions on the surface of the working electrode under the influence of an electric field. The shape and rate of crystal formation can be changed by adjusting the voltage, current, and concentration of the reaction solution. For instance, Yang et al. [76] created a nickel film superhydrophobic surface by depositing nickel onto a copper surface using a one-step electrodeposition process. As shown in Figure 7a, a homogeneous electrolyte was made from a mixture of nickel chloride (NiCl_2_–6H_2_O) and myristic acid (CH_3_(CH_2_)_12_COOH), where myristic acid served as a surface modifier. When an electric current was applied, nickel ions (Ni^2+^) moved to the cathode electrode and gained electrons to produce nickel (Ni). In addition, nickel ions (Ni^2+^) reacted with myristic acid (CH_3_(CH_2_)_12_COOH) to form nickel myristate (Ni[CH_3_(CH_2_)_12_COO]_2_), which formed low surface energy functional groups on the cathodic electrode surface. Meanwhile, some hydrogen ions (H^+^) around the cathode plate also gained electrons to form hydrogen (H_2_) during the reaction process, the released hydrogen gas caused the surface of the cathode electrode to be loosened. The above reactions resulted in a nickel film superhydrophobic surface with a rough structure such as cauliflower or thorn, as shown in Figure 7b,c.

Similarly, Li et al. [77] used electrochemical deposition to gain a hydrophobic ZnO film. Then, the superhydrophobic ZnO film was gained with (fluoroalkyl) silane modification; the electrochemical settings may be adjusted to alter the film’s thickness and morphology. Shirtcliffe et al. [78] electrodeposited copper ions from an acidic copper sulfate solution onto the surface of a copper plate by electrochemical deposition, forming a rough surface. Then, superhydrophobic surfaces were obtained after heat treatment and fluorination. To create superhydrophobic surfaces with high contact and low rolling angles, Su et al. [79] performed Ni electrodeposition in a typical Watts bath containing NiSO_4_, NaCl, and H_3_BO_3_. Then, the superhydrophobic surface was obtained by heat treatment and fluorination. Remarkably, the superhydrophobic surfaces may be employed in both alkaline and acidic conditions and exhibit good hardness and wear resistance.

Electrochemical deposition methods have the advantages of being a simple operation and having a low cost, which provides a viable approach to the preparation of superhydrophobic coatings for a variety of metallic materials and has broad industrial application prospects.

#### 3.2.4. Chemical Vapor Deposition

Chemical vapor deposition is a technique in which gas reactants are deposited on a solid surface to form a thin film. Notably, the morphology of the film on the solid surface can be altered by changing the type of gas reactant and the reaction conditions.

Huang et al. [80] used chemical vapor deposition to synthesize carbon nanotubes on Si substrates and deposited alumina on carbon nanotubes to obtain stable superhydrophobic surfaces. Among them, carbon nanotubes provide the nanostructure, and the presence of ZnO makes the surface have low surface energy. Notably, the wettability of the ZnO–CNTs surface can be modified by UV irradiation and light-free storage. To further reduce the operational difficulty of the chemical deposition method, Zimmermann et al. [81] prepared a silicon nanowire superhydrophobic textile coating by a one-step chemical deposition method. The textile superhydrophobic coating had good superhydrophobic properties. When the textile coating was slightly tilted, the water droplets on the coating rolled off. In addition, the coating had good mechanical stability. After 1450 wear cycles, the textile’s superhydrophobic coating still had superhydrophobic properties.

#### 3.2.5. Other Methods of Generating Coatings

There are also sol-gel and self-assembly techniques for creating superhydrophobic coatings on surfaces in addition to the three ways mentioned above. The fabrication of superhydrophobic coatings using the sol-gel technique involves two key steps: (1) a highly chemically active compound is used as a precursor, which undergoes a hydrolytic condensation reaction to form a sol in solution; (2) the resulting sol slowly polymerizes between colloidal particles to form gels, and when the solvent has completely evaporated, a micro- and nanostructured surface is formed [68,82]. For instance, Nadargi et al. [83] used the sol-gel technique to create a superhydrophobic silica-iron oxide nanocomposite. Methyltrimethoxysilane (MTMS) was hydrolyzed for 24 h to create a sol, and aqueous iron nitrate and aqueous ammonium hydroxide were added for stirring to cause significant aggregation of the MTMS sol to form a gel; the gel was then aged in methanol for two days to reinforce the gel network.

Self-assembly, a process that turns elements of an already-existing chaotic system into an ordered molecular whole without the need for human intervention, is a method for creating covalent bonds between atoms to create molecules. Liu et al. [84] prepared a novel smart stimuli-responsive superhydrophobic coating by self-assembly of graphene monolayers and titanium dioxide nanofilms on a substrate. Notably, UV irradiation can change the wettability of this superhydrophobic coating; this was because when the superhydrophobic coating was irradiated by UV light, the atomic structure of titanium dioxide was changed, producing particles and particle clusters with greater surface energy. The design of this coating offers crucial suggestions for future stimulus-responsive surfaces.

### 3.3. Etching Method

The etching method mainly uses lasers or chemical reagents to etch substrates to build superhydrophobic surfaces with micro- and nanostructures [85,86,87]. The method can build the rough structure directly on the solid surface, so there is no need to consider the adhesion between the coating and the substrate interface, and it is promising to build the rough structure and obtain the superhydrophobic surface by etching directly on the metal surfaces [4].

#### 3.3.1. Chemical Etching Method

Common metals such as copper and aluminum have many dislocation defects that are high in energy and easily destroyed. Therefore, when the metal is exposed to a chemical etchant, the dislocation defects with greater energy react preferentially and generate a rough structure [88]. Chemical etching is the process of building rough structures on solid surfaces using chemical reagents to create superhydrophobic surfaces. Acidic and alkaline solutions are often used as etchants.

**Figure 8:**
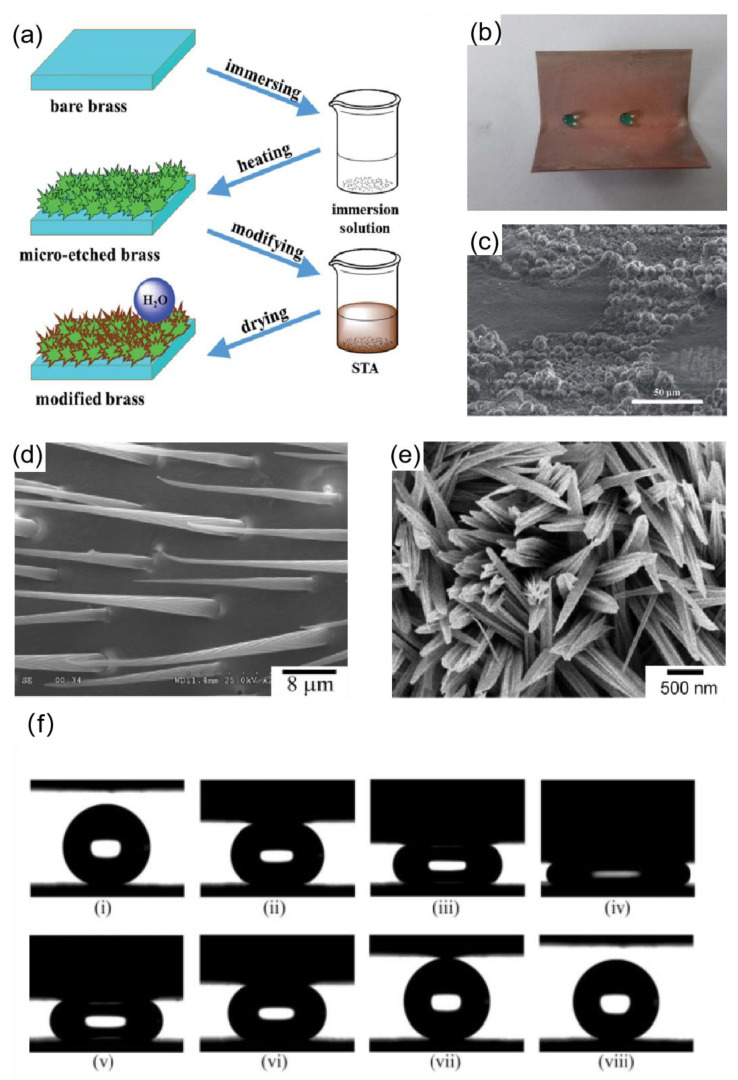
(**a**) Process flow diagram for preparing superhydrophobic surfaces on copper plates [89]. (**b**) Optical photographs of the shape of the water drop at the bend of the copper plate [89]. (**c**) SEM image of the bending copper plate area [89]. (**d**) SEM image of the setae on a water strider’s leg. (**e**) SEM image of Cu(OH)_2_ superhydrophobic surfaces [92]. (**f**) Sequential snapshots of two superhydrophobic surfaces undergoing compression and relaxation processes. Image (iv) shows the maximum compression between the two surfaces. In image (vii), the water droplet was completely separated from both surfaces. The whole sequence appears to be reversible [92].

Common acids such as nitric acid and hydrochloric acid are widely applied as etchants in chemical etching. Tan et al. [89] used micro-etching techniques to construct micro and nano rough structures on brass surfaces, which were modified by stearic acid to reduce surface energy. First, to remove surface oxides, polished and cleaned brass plates were submerged in a solution of 10 wt% H_2_SO_4_ solution for 30 s. NaCI and Na_2_SO_4_ were added to a reaction solution containing 1 M HCI. Brass plates that had undergone the aforementioned treatment were submerged in the prepared solution for 10 s before being removed, heated in an oven, and micro-etched to create a rough surface structure. The samples were immersed in an ethanol solution of 0.1 M stearic acid (STA) for 2 min. Finally, a superhydrophobic surface was obtained, as shown in Figure 8a. The superhydrophobic surface had a contact angle of 152.4°, and when the sample was bent at a 60° angle, only a small portion of its micro-nanostructure was lost, as shown in the Figure 8b,c; the majority of it remained the same, so the water droplets at the bend remained spherical. A similar strategy was utilized by Qu et al. [90], who used a mixture of nitric acid and hydrogen peroxide as an etchant and fluoroalkylsilanes to reduce the surface energy. Wang et al. [91] created a flowerlike cluster of a superhydrophobic surface on copper plates by soaking copper plates in an ethanolic solution of n-tetra decanoic acid for three days. The flowerlike cluster superhydrophobic surface had a contact angle of up to 162°, and the rolling angle was only 2°.

**Figure 9:**
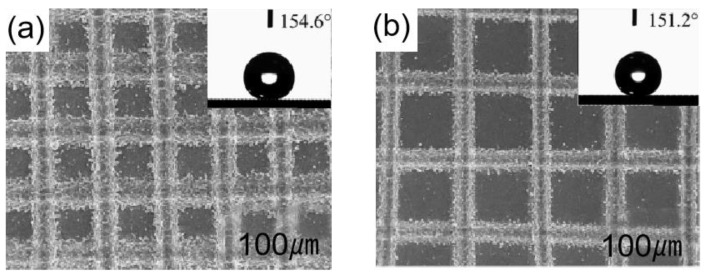
SEM micrographs of the laser-processed surface: (**a**) images for a line spacing of 150 μm; (**b**) images for a line spacing of 200 μm. The insets in (**a**,**b**) illustrate the corresponding contact angles after laser processing [94].

Alternatively, common alkaline solutions can be used for chemical etching. For instance, inspired by the hierarchical structure of a water strider’s leg, Yao et al. [92] prepared a surface with a conical nanoarray structure similar to the leg structure of a water strider by submerging the copper surface in an ammonia solution. After surface modification, superhydrophobic surfaces of copper hydroxide were obtained, as shown in Figure 9d,e. It was also worth noting that, unlike other superhydrophobic surfaces, the droplets could recover from the Wenzel state to the Cassie–Baxter state when two identical copper hydroxide superhydrophobic surfaces were subjected to steps such as squeezing and relaxing the droplets; this was primarily because the conical nanoarray structure had lateral nano-slots that released the fixation of the deformation boundary, as shown in Figure 9f.

Guo et al. [93] used wet chemical etching to obtain rough structures on the surface of aluminum alloys and modified them to obtain superhydrophobic surfaces. To create the superhydrophobic surfaces, aluminum alloys were submerged in various concentrations of NaOH solution for several hours. After being rinsed with deionized water to remove any remaining surface impurities, the alloys were then annealed at 120 °C for about an hour, modified with perfluorononane, and then dried at 80 °C for two hours. The contact angle of the surface reached 157°, and the superhydrophobic properties of the surface were affected by the concentration of the NaOH solution and the roughness of the substrate surface.

#### 3.3.2. Laser Etching Method

In laser etching, when the material surface is irradiated by laser, the material surface will be ablated and melted, thus forming a rough surface. The morphology of the surface microstructure is influenced by the energy density of the laser beam, the scan rate, and the scan spacing. The issue of the unequal distribution of rough structures on superhydrophobic surfaces can be resolved by laser etching, and superhydrophobic structures can be precisely constructed. Additionally, high-hardness materials can still be etched with a laser.

Li et al. [94] used one-step laser processing to create lattice structures on aluminum alloy surfaces. When the surfaces were irradiated, different lattice types could be created by adjusting the laser’s power and line spacing. The splash structure, lattice structure, and groove structure generated throughout the operation were primarily what provided this sample its superhydrophobic qualities. This surface reached a contact angle of 154.6° and a roll angle of 3°. As shown in Figure 9a,b, the contact angle decreases as the line spacing increases from 150 m to 200 m during laser beam irradiation.

**Figure 10:**
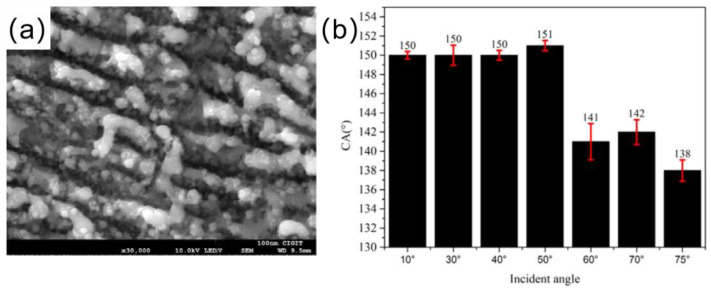
(**a**) SEM image of a femtosecond laser incidence angle of 50° [95]. (**b**) Histogram of titanium surface contact angle versus femtosecond laser incidence angle [95].

Shen et al. [95] created microstructures and nanostructures on the titanium surface by obliquely irradiating femtosecond laser onto a surface, as shown in Figure 10a. As shown in Figure 10b, the surface contact angle also changed as the laser beam incidence angle did, this was mainly because different incident angles of the laser beam create different rough structures. When the laser beam incident angle was 50°, the contact angle on the titanium surface was 151°.

However, laser etching for the large-scale preparation of superhydrophobic surfaces has not been studied enough. When a laser is used to treat non-metallic materials, it leads to deformation and changes in the surface chemistry of the non-metallic material. In addition, the equipment used for laser etching is more expensive, resulting in higher production costs.

## 4. Advances in Research on Superhydrophobic Surfaces

In recent years, the ability of superhydrophobic coatings to delay and reduce ice formation has been explored and studied by researchers [43]. The continuous development of superhydrophobic materials has shown good prospects for application in many fields. An increasing number of studies have shown that superhydrophobic materials have the characteristics of delayed icing and reduced ice adhesion [52,96,97,98,99,100,101]. However, the anti-icing capability of superhydrophobic materials is affected by many factors, such as low-temperature failure, fragile micro-nanostructured surfaces, and low durability. Therefore, to address these issues, researchers have developed superhydrophobic coatings such as wear-resistant and self-healing surfaces [102,103,104]. Meanwhile, to further improve the de-icing ability of superhydrophobic surfaces, the idea of combining photothermal or electrothermal effects with superhydrophobic coatings has been proposed by researchers. Research progress of wear-resistant superhydrophobic coatings, self-healing/self-repair superhydrophobic coatings, photothermal superhydrophobic coatings, and electrothermal superhydrophobic coatings in the field of anti-icing and de-icing are highlighted in this section.

### 4.1. Superhydrophobic Coating Based on Passive Anti-Icing

Ordinary superhydrophobic coatings often suffer from a lack of mechanical stability. When the coating is abraded by external forces, its micro-nanostructure is destroyed, leading to the failure of the superhydrophobic coating. Therefore, the development of a superhydrophobic coating with wear-resistant, self-healing, or self-healing effects is the key to solving this problem.

#### 4.1.1. Wear-Resistant Superhydrophobic Coating

Superhydrophobic biomimetic coatings are excellent at preventing ice [38,41,105]. However, superhydrophobic coatings are challenging to use in practice due to their fragile microstructures and nanostructures and lack of sustained stability [106]. Therefore, the development of superhydrophobic coatings with high mechanical stability is the key to solving this problem.

**Figure 11:**
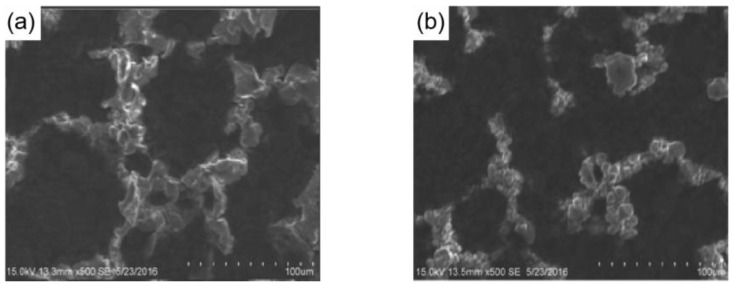
(**a**) SEM image of the PTFE/PDMS superhydrophobic coating with 0.6 g of PTFE added [107]. (**b**) SEM image of the surface after being de-iced 34 times [107].

Researchers have improved the mechanical stability of superhydrophobic coatings by adding some flexible polymers. For example, Ruan et al. [107] mixed polytetrafluoroethylene (PTFE), polydimethylsiloxane (PDMS), and tetraethyl orthosilicate to prepare a PTFE/PDMS superhydrophobic coating by spin coating on an aluminum substrate. When 0.6 g of PTFE was added, the contact angle of the coating was as high as 163.6°. As shown in Figure 11, the surface structure of the coating remained essentially unchanged after 34 icing/de-icing cycles and the coating exhibited good mechanical wear resistance after 1 m of mechanical abrasion with sandpaper. When the temperature dropped from 13.3 °C to −6 °C, the water droplets on the aluminum plate completely froze in only 37 s, while the water droplets on the superhydrophobic coating took 82 s before ice nuclei started to form, thus demonstrating the ability of the PTFE/PDMS superhydrophobic coating to delay icing.

Using surface covering meshes and spraying silica nanoparticles on the outer surface, Kravanja et al. [108] successfully prepared superhydrophobic concrete samples with excellent mechanical stability by adding hydrophobic non-fluorinated additives to the inner surface. The sample exhibited good superhydrophobic properties with contact angles of up to 157.6° and rolling angles of up to 6.5° and maintained superhydrophobic properties under high-intensity mechanical wear, with six times lower de-icing strength compared to the unmodified surface.

**Figure 12:**
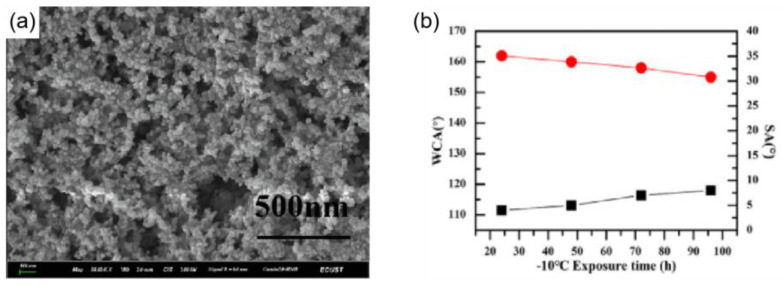
(**a**) FE-SEM image of the F–PU/SiO_2_ surface spin-coated with 2 wt% SiO_2_ solution [109]. (**b**) Variation of WCA and SA of the coating with time at −10 °C [109].

Lv et al. [109] prepared a wear-resistant and ice-resistant superhydrophobic surface by bonding nano-SiO_2_ to a polyurethane surface under light-curing conditions, as shown in Figure 12a. When the silica content was 2 wt%, the superhydrophobic surface showed excellent superhydrophobicity (CA ≈ 165°, SA ≈ 2°) and good mechanical stability. In a −10 °C environment, water droplets on a blank aluminum sheet froze after 6 min, while on this coating, water droplets completely froze after 56 min, which is a result that shows that the superhydrophobic coating had good delayed icing, and the superhydrophobicity remained when left at −10 °C for 100 h, as shown in the Figure 12b. The superhydrophobicity also remained after 150 cycles of rubbing with sandpaper under an applied load of 100 g. Additionally, by creating covalent connections between the coating and the substrate or by cross-linking with the coating, the superhydrophobic coatings’ mechanical stability can be increased [103].

#### 4.1.2. Self-Repairing/Self-Healing Superhydrophobic Surfaces

The application of superhydrophobic materials is significantly limited by the fact that the micro and nano rough structure of the surface is easily damaged by external scratching, while the modified low surface energy substances can be decomposed in harsh environments, resulting in the loss of superhydrophobicity of the material. To overcome this problem, some researchers have combined self-healing properties with superhydrophobicity to produce self-healing superhydrophobic materials with excellent surface stability. When the self-repairing/self-healing superhydrophobic surfaces lose superhydrophobicity, the superhydrophobicity can be repaired spontaneously or under certain conditions. Therefore, the study of self-healing superhydrophobic materials is of great importance both from the point of view of scientific theory and practical application.

Fu et al. [110] synthesized a new fluorinated polyurethane (FPU) on a substrate by a two-step thiol click reaction and then added SiO_2_ nanoparticles to prepare a robust superhydrophobic surface with self-healing capabilities. The polyurethane chains had a greater tendency to move into the coating, while the fluoroalkyl chains tended to stay on the surface of the coating or had a tendency to migrate to the upper surface. Therefore, when the surface was damaged, the fluoroalkyl chains migrated to the upper surface by heating at 130 °C for 1 h and regained their superhydrophobic properties, achieving a self-healing effect. In addition, the coating had a good delayed-icing capability, with water droplets on the coating completely icing after 504 s at −20 °C; the icing time of the coating was seven times of the bare substrate.

**Figure 13:**
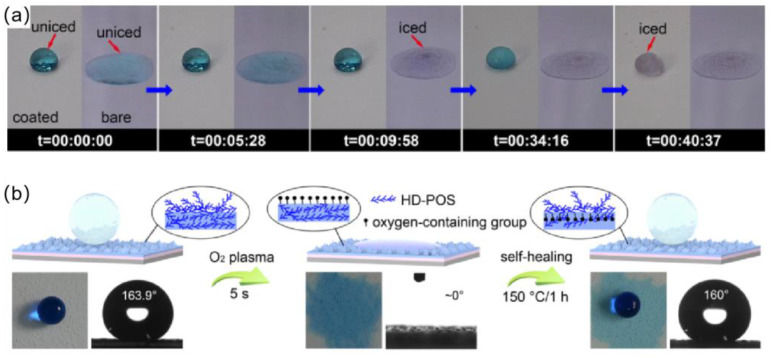
(**a**) Photographs of the icing process of water droplets on the superhydrophobic coating and bare glass slide at −15 degrees Celsius [111]. (**b**) Schematic illustrations of the mechanism and process of damaged/self–repair of the PU/SiO_2_@HD–POS superhydrophobic coating, and the corresponding photographs of water droplets on the coatings [111].

However, preparation of most self-healing superhydrophobic coating involves the use of fluorides and organic solvents, and there is a chance that the environment could be contaminated. Therefore, Li et al. [111] first reported a robust self-healing superhydrophobic coating that was completely aqueous and without fluoride involvement. The coating was obtained by successively spraying polyurethane (PU) aqueous solution and a cetyl polysiloxane-modified SiO_2_ aqueous suspension onto the substrate. Unlike other superhydrophobic coatings, the surface of this coating had large protrusions that form a macroscopically rough structure. When sandpaper abrasion and tape peeling tests were conducted, the coating showed good mechanical stability, which was attributed to its macroscopic rough structure and the polyurethane utilized as a binder. As shown in the Figure 13a, water droplets on the self-healing superhydrophobic coating completely froze in 40 min 37 s at −15 degrees Celsius, while water droplets on the bare substrate completely froze in 9 min 58 s, and the coating did not freeze when water droplets were continuously dropped on the tilted coating. The above results indicated the coating’s good anti-icing ability. In addition, the coating was also self-healing, and when the coating’s surface was damaged by O_2_ plasma, the surface transformed from superhydrophobic to superhydrophilic. However, after heating at 150 °C for 1 h, the coating regained its original superhydrophobic properties because cetyl groups migrated to the coating’s upper surface during the heat treatment, and hydrophilic groups were embedded in the coating, as shown in Figure 13b.

In addition, superhydrophobic coatings with similar chemical composition and structure can be constructed on the exterior and interior of the surface. The superhydrophobic coating can be repaired by simply rubbing the damaged area away to reveal a fresh coating with the same structure [112].

However, wear-resistant superhydrophobic coatings and self-healing/self-repairing superhydrophobic coatings still generate ice in harsh environments, which causes the failure of superhydrophobic coatings. This is a major challenge for superhydrophobic coatings in the field of anti-icing. To solve the above problem, researchers proposed the idea of combining passive anti-icing with active de-icing.

### 4.2. Superhydrophobic Coating Based on a Combination of Passive Anti-Icing and Active De-Icing

The anti-icing properties of superhydrophobic coatings have been demonstrated, but there are still differing views on whether superhydrophobic coatings can reduce ice adhesion. Some researchers believe that a superhydrophobic coating can reduce ice adhesion [96,98,100,101], but others believe that ice adhesion strength increases when superhydrophobic coating icing occurs, which is mainly due to the anchor effect caused by water condensation in the microstructures and nanostructures of superhydrophobic coatings in wet or cold environments [113,114]. Ice formation in the surface microstructure can damage the superhydrophobic coating microstructure, leading to the failure of the anti-icing properties of the superhydrophobic coating [115,116,117]. To solve this issue, an idea to combine photothermal materials with superhydrophobic coatings has been proposed. Passive anti-icing performance is accomplished using the superhydrophobic surface, while active de-icing performance is usually accomplished using photothermal and electrothermal effects.

#### 4.2.1. Photothermal Superhydrophobic Coating

Solar energy as nature’s largest green renewable energy source has a lot of potential for application in the field of de-icing. The use of the photothermal effect can delay the formation of ice or melt it directly, but the water that remains on the surface after melting absorbs the temperature generated by the photothermal surface, which slows down the melting rate of the unmelted part of the ice; therefore, an idea to embed photothermal materials in superhydrophobic coatings has been proposed by researchers. The photothermal superhydrophobic coatings melt the ice using the photothermal effect, and then, melted water droplets will roll out of the coating due to superhydrophobicity, preventing any impact on the unmelted ice [118]. Even at night, the photothermal superhydrophobic coatings can also reduce or delay ice formation. Common photothermal materials include semiconductor materials (e.g., copper sulfide, zinc oxide, etc.) [119], carbon materials (e.g., graphite, graphene, graphene oxide, carbon nanotubes, carbon black, etc.) [120,121,122], precious metal nanoparticles (e.g., gold, silver, etc.) [123], bio-melanin [124], etc.

**Figure 14:**
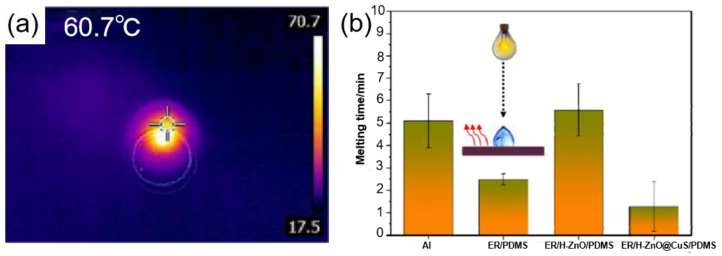
(**a**) Thermal infrared imaging of H-ZnO@CuS [119]. (**b**) Ice melting time for different composite coatings [119].

Some semiconductor materials have the capability of photothermal conversion. When a semiconductor material is irradiated by light with energy equal to or higher than the band gap, electron-hole pairs are generated in the semiconductor and the excited electrons eventually return to lower energy levels. Releasing energy in the form of photons [125], or by non-radiative relaxation, the excited electrons transfer energy to the impurity/defect, and the energy is released into the crystal system in the form of phonons, causing local heating of the lattice, thereby generating heat [126,127]. Bao et al. [119] prepared an ER/H-ZnO@CuS/PDMS photothermal superhydrophobic coating with excellent superhydrophobic properties and mechanical durability by using hydrangea-like ZnO@CuS (H-ZnO@CuS) as photothermal nanofiller particles. As shown in Figure 14a, after being irradiated by an 808 nm laser, the temperature of the hydrangea-shaped ZnO@CuS (H-ZnO@CuS) rapidly increased to 60.7 °C, indicating that the hydrangea-shaped ZnO@CuS (H-ZnO@CuS) had good photothermal properties. At −15 °C, the freezing time of water droplets on this photothermal superhydrophobic surface was 4.3 times longer than that of pure aluminum. As shown in Figure 14b, even if water droplets froze on the surface, when the photothermal superhydrophobic coating was illuminated, the surface melted the droplets within 1.5 min. It is worth noting that after 40 friction cycles or 100 tape peels (tape adhesion of 0.44 N/mm) the photothermal superhydrophobic coating still had good superhydrophobic properties.

**Figure 15:**
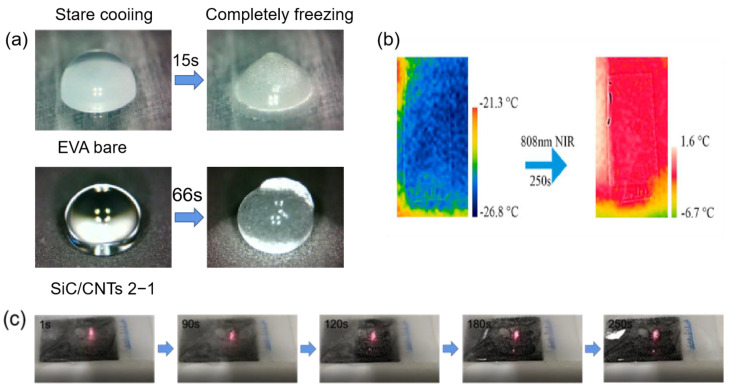
(**a**) Optical photographs of water droplets starting to freeze and completely freezing on EVA bare and SiC/CNTs 2−1 surfaces [130]. (**b**) Infrared thermography images of the superhydrophobic coating surface before and after photothermal deicing [130]. (**c**) Optical images of the photothermal deicing process on coating surfaces [130].

Carbon materials also have a photothermal conversion effect. Broadband light can be efficiently absorbed by carbon-based materials. When light is illuminated on the carbon materials, π-orbiting electrons are excited to π* orbitals and electron leaps occur; the light-absorbing electrons rise from the ground state (HOMO) to a higher energy orbital (LUMO), releasing energy in the process [128]. Through electron–phonon interactions, the absorbed light energy is transmitted from excited state electrons to vibrational modes throughout the lattice, raising the material’s surface temperature [126,129]. Jiang et al. [130] prepared a photothermal SiC/CNTs superhydrophobic coating by using ordinary cable sheath material (EVA) as a substrate and spraying carbon nanotubes as photothermal filler particles. When the ratio of SiC to CNTs was 2:1, the contact angle could reach 161° and the roll angle was less than 2°, and it had excellent anti-icing properties. As shown in Figure 15a, when at −30 °C, the freezing time of water droplets on the SiC/CNTs superhydrophobic coating was 66 s, which was 4.4 times that of the EVA surface. In addition, the SiC/CNTs photothermal superhydrophobic coating also had excellent de-icing capabilities. When the SiC/CNTs coating was exposed to NIR (808 nm, 2.5 W) for 10 s, the coating temperature increased to approximately 120 °C, while there was no change in temperature on the EVA surface. As shown in Figure 15b,c, carbon nanotubes (CNTs) had excellent photothermal conversion properties and thermal conductivity; when a large area of ice on the coating was irradiated by NIR (808 nm, 1 W), the generated heat was transferred from the illuminated area to the surrounding coating and the average temperature of the coating rose to −2.4 °C, resulting in the melting of the ice. The method is of great importance for large-area anti-icing and de-icing.

**Figure 16:**
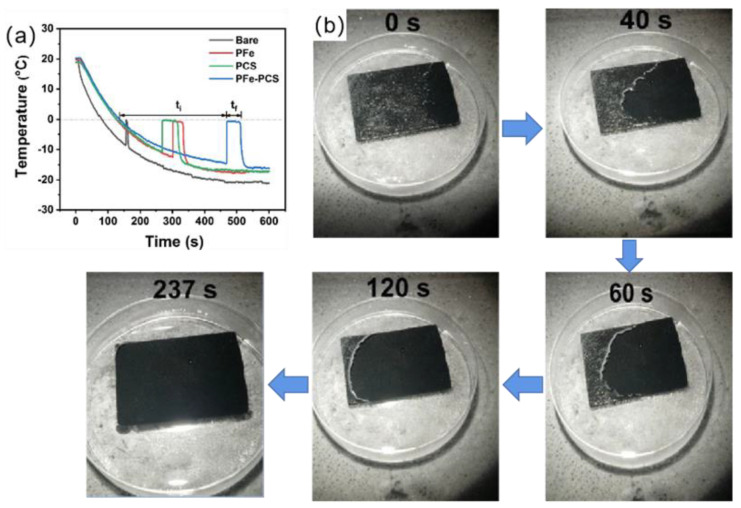
(**a**) Temperature profiles of water droplets on bare, PFe, PCS, and PFe−PCS surfaces [131]. (**b**) Photothermal de-icing process of a thin ice layer of approximately 1 mm thickness on PFe−PCS under 1 solar irradiation [131].

Zheng et al. [131] prepared a magnetically responsive and soft superhydrophobic photothermal film consisting of polydimethylsiloxane (PDMS), iron powder (Fe), and candle soot (CS), where the candle soot (CS) acts as a photothermal particle. The superhydrophobic photothermal film was formed by superimposing a PDMS/Fe superhydrophobic layer and a PDMS/CS superhydrophobic layer. This structure created two air layers, which prevented heat from escaping from the photothermal superhydrophobic film during illumination. Due to the presence of iron powder (Fe), the superhydrophobic film had magnetic responsivity and could fit tightly to complex substrates under a magnetic field. As shown in the Figure 16a, PFe−PCS film had good delayed-icing performance relative to other coatings when the temperature ranged from 20 °C to −20 °C. The total freezing time for the bare surface, the PFe coating, and the PCS coating were 81.3 s, 214.5 s, and 193.9 s, respectively, while the summary ice time for the PFe−PCS film was up to 385.2 s; this is because the PFe−PCS film had a double-layer micro-nanostructure. Total freezing time was calculated as t = t_i_ + t_f_, where t_i_ was the time required for the droplet to go from 0 °C to the phase transition temperature and t_f_ was the time required for the droplet to start the phase transition to freeze completely. As shown in the Figure 16b, an ice layer of 1 mm on the PFe−PCS film was melted completely at 237 s when irradiated with 1 solar light at −5 °C. In addition, the photothermal superhydrophobic film had good durability and resistance to acids and alkalis.

**Figure 17:**
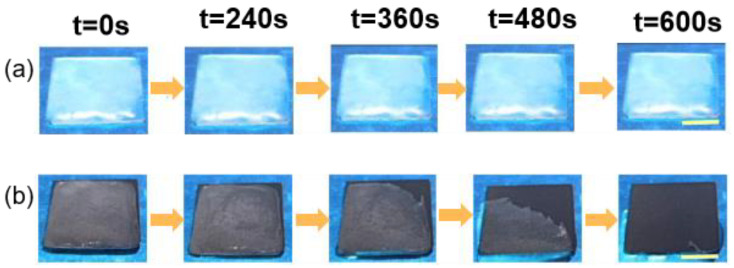
Optical images of photothermal deicing processes on different surfaces under one solar intensity. (**a**) Uncoated glass; (**b**) superhydrophobic photothermal coating [124].

Xue et al. [124] extracted melanin nanoparticles from cuttlefish juice and prepared a photothermal superhydrophobic coating using melanin nanoparticles, modified SiO_2_ nanoparticles, and PDMS precursors, where the melanin nanoparticles were used as photothermal filler particles. The freezing time of water droplets on an aluminum plate at −20 °C was 30 s, while the freezing time on the photothermal superhydrophobic coating was 144 s, which was five times longer than the freezing time of the aluminum plate. This difference can be attributed to the micro-nanostructure of the photothermal superhydrophobic coating, which increased the liquid–gas contact area and decreased the heat transfer from the water droplets to the colder surface. As shown in Figure 17, when the coating was illuminated after icing, the ice on the photothermal superhydrophobic coating gradually melted, and, eventually, the melted water droplets rolled off the coating to achieve a de-icing effect, and there was no change in the ice on the bare surface.

In addition, precious metal materials can also generate heat when they are illuminated by light. Metal nanoparticles can absorb light in a specific wavelength range when infrared light strikes precious metal materials, producing a plasma resonance effect (LSPR) [132]. Hot electrons are excited from occupied to non-occupied states under the effect of the resonance effect (LSPR), and, due to electron–electron interactions and electron–phonon interactions, the energy can be converted into vibrational energy of the crystal lattice and transferred to the surrounding medium, thus increasing the surface temperature [133]. However, precious metals are significantly more expensive than the photothermal materials listed before, so it is not suitable for large-scale preparation of photothermal superhydrophobic coatings.

#### 4.2.2. Electrothermal Superhydrophobic Coating

Traditional electrothermal de-icing has many shortcomings, such as causing fires and consuming large amounts of energy. Therefore, the idea of combining electrothermal effects with superhydrophobic properties, which uses lower voltage to generate heat to prevent fires caused by high voltage and significantly reduces energy consumption, has been proposed by researchers. The combination of superhydrophobic coatings and electrothermal properties offers a promising strategy for durable anti-icing and de-icing [134].

**Figure 18:**
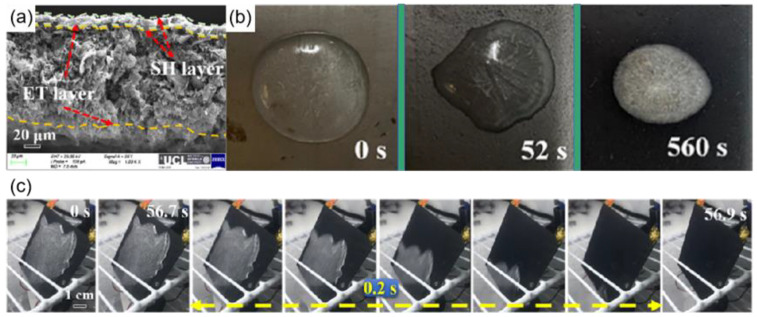
(**a**) Fracture surface of SH@ET nanocomposite coating [136]. (**b**) Ice formation time of 2 mL of water in stainless steel, ET coating, and SH coating, respectively [136]. (**c**) Ice melting after powering the SH@ET coating [136].

**Figure 19:**
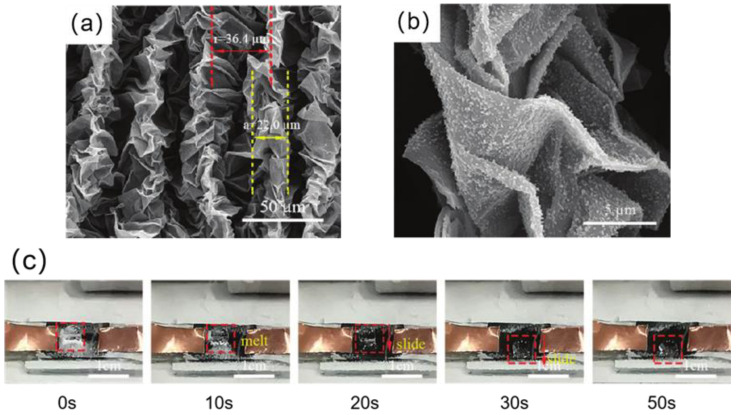
(**a**) SEM images of SiO_2_/rGO superhydrophobic folded film. The size of the wrinkles and the distance between them are indicated by the yellow dashed line and the red dashed line, respectively [138]. (**b**) High magnification SEM image of SiO_2_/rGO superhydrophobic folded film. The size of the wrinkles and the distance between them is shown by the yellow dashed line and the red dashed line, respectively [138]. (**c**) Electrothermal de-icing process of SiO_2_/rGO superhydrophobic folded film [138].

Conductive nanomaterials with high heating rates and ease of use are ideal for preparing electrothermal superhydrophobic coatings [135]. Among them, graphene, carbon nanotubes, and reduced graphene oxide are the more commonly used conductive nanomaterials. Hou et al. [136] obtained an electrothermal superhydrophobic coating by combining an electrothermal (ET) layer with a superhydrophobic layer (SH). Graphene with good electrical conductivity was used as the lower electrothermal layer, and carbon nanotubes (CNTS) and SiO_2_ were used as the upper superhydrophobic layer, as shown in Figure 18a. The resistances could be adjusted by changing the thickness of the graphene electrothermal layer. The electrothermal superhydrophobic surface had a contact angle of up to 163° and a rolling angle of 2°. As shown in Figure 19b, the freezing time of 2 mL of water on the SH@ET coating (560 s) was much greater than that for the stainless steel (0 s) and ET coatings (52 s), and larger ice crystals were formed on the SH@ET coating after the water droplets froze; this was because the superhydrophobic surface had air layers, which hindered the heat transfer and delayed the freezing time. Additionally, the SH@ET coating had good de-icing capabilities; when the coating was energized, it caused the ice on the coating to melt after 60 s due to the effect of Joule heat, and water droplets didn’t remain on the coating, as shown in Figure 18c.

Wang et al. [137] developed an electrothermally driven shape-memory composite structure with tunable wettability, which was prepared by a superhydrophobic layer with micro-pillars of MWCNTs/ESMP and adhered to an electrothermal layer of MWCNTs/PDMS. Due to the good electrothermal properties of the multi-walled carbon nanotubes (MWCNTs), the electrothermal superhydrophobic coating heated up rapidly to 140 °C at 12 V. This electrothermal superhydrophobic coating had good anti-icing properties. Compared to a smooth surface, the start of electrothermal superhydrophobic coating freezing time was delayed for 199 s and the complete freezing time was delayed for 256 s. Notably, the shape memory and the fast electrothermal response capability of the MWCCNTs/ESMP layer allowed the surface micropillar array to switch between deformed and upright states, resulting in changes in surface-wetting properties. The design concept provided ideas for dynamic anti-icing. When in a −10 °C environment, this electrothermal superhydrophobic surface had good anti-icing properties, with the start of freezing time being delayed by 199 s and the complete freezing time being delayed by 256 s compared to a smooth surface.

Chu et al. [138] used reduced graphene oxide as the electrothermal material to prepare a SiO_2_/rGO superhydrophobic folded film modified by 1H, 1H, 2H, 2H-perfluoro decyl trichlorosilane (FDTS). As shown in Figure 19a,b, due to the existence of folds and SiO_2_, the film had a micro-nanostructure. When the folded size is 22 μm and the fold spacing is 36.4 μm, the electrothermal superhydrophobic film’s contact angle can reach 161.4° and its rolling angle can be as low as 1.2°. The superhydrophobic electrothermal film delayed the freezing time of water droplets by 297 s at −10 °C, which was 8.3 times longer than that of the smooth rGO film. In addition, after passing a DC voltage of 15 V, the superhydrophobic electrothermal film can melt the ice accumulation in 30 s in a −20 °C environment, as shown in Figure 19c.

However, superhydrophobic coatings are easily scratched or even broken under conditions of external forces in complex and changing environments, which greatly reduces the anti-icing effect of superhydrophobic coatings and shortens the service life of superhydrophobic coatings. Therefore, Peng et al. [139] prepared a stretchable electrothermal superhydrophobic coating by embedding hydrophobically modified graphene into Ecoflex elastomer. Due to the resilient properties of Ecoflex, the coating was able to maintain excellent superhydrophobic properties even under conditions of large deformation.

**Figure 20:**
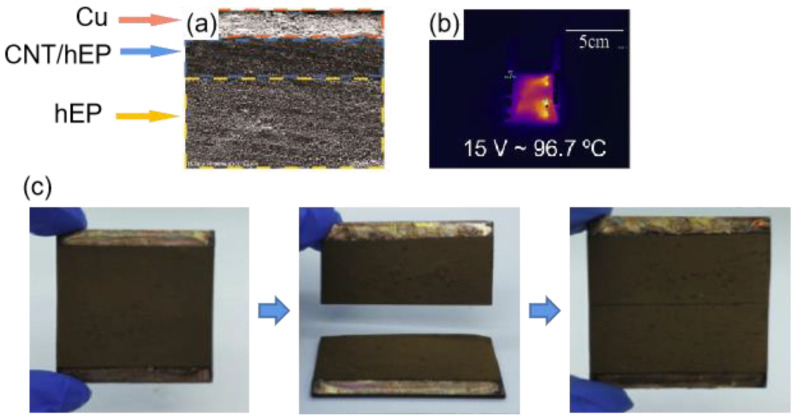
(**a**) Layered structure of the coating [140]. (**b**) Maximum surface temperature of nCu-CNT3/hEP-hEP at 15 v. (**c**) Self-healing process of the nCu-CNT3/hEP-hEP coating [140].

To further improve the durability of electrothermal superhydrophobic coatings, Li et al. [140] prepared an electrothermal superhydrophobic three-layer composite with high self-healing capabilities after complete rupture based on the self-healing epoxy resin (hEP), multi-walled carbon nanotubes (CNTs), and superhydrophobic copper powder (nCu), as shown in Figure 20a. The microstructures and nanostructures formed by the superhydrophobic copper powder can trap air, thus making a composite superhydrophobic coating with a contact angle of up to 154° and a roll angle as low as 3°. As shown in Figure 20b, the nCu-CNTs/hEP-hEP composite can reach a temperature of 96.7 °C when a voltage of 15 V is applied to it. As shown in Figure 20c, the nCu-CNTs/hEP-hEP composite coating was cut into two pieces and the two coatings were in contact with each other and placed at 160 °C for 1h. Finally, a perfect composite block was obtained, and the healed composite coating also showed good superhydrophobicity and anti-deicing properties, which is mainly due to the good ductility and self-healing properties of hEP. The proposed composite material greatly improved the efficiency of the electrothermal anti-deicing coating for anti-icing.

## 5. Summary and Outlook

In this paper, three models of the wetting phenomenon on solid surfaces are introduced. Then, the main principles of anti-icing on superhydrophobic surfaces and methods for preparing superhydrophobic surfaces were summarized. Current methods for the preparation of superhydrophobic coatings are usually costly, use harmful chemicals, and are difficult to scale up [141,142]. To make superhydrophobic coatings widely available, researchers should develop advanced techniques for fabricating superhydrophobic materials, which should be easy to operate, cost-effective, and environmentally friendly and greatly facilitate the large-scale preparation of superhydrophobic materials. In addition, the advantages and disadvantages of conventional anti-icing methods and superhydrophobic anti-icing methods were summarized as shown in Table 2.

Compared with traditional anti-deicing techniques, superhydrophobic coatings can roll off the surface before the water droplets freeze. Due to superhydrophobic coatings having lower surface energy and a smaller solid–liquid contact area, they can enhance the free energy barrier of ice cores and restrict the heat energy exchange from droplets to colder surfaces, which in turn delays the freezing period of water droplets. In addition, due to the presence of microstructures and nanostructures on the superhydrophobic coatings, when the superhydrophobic coatings have ice formation, the contact area between the ice and the coatings becomes smaller, making the adhesion of ice to the coatings lower and easier to remove. However, it is also suspected that the superhydrophobic coatings can increase the adhesion of ice; this is due to the anchor effect between the ice and the coating.

It is generally known that a superhydrophobic coating’s performance is mostly influenced by its surface topography. However, the microscopic morphology of a superhydrophobic coating is very easily disrupted, which leads to the failure of the superhydrophobic coating. Excellent Cassie state stability, high water resistance, minimal ice adhesion, and strong mechanical wear resistance are necessary for good superhydrophobic coatings [35]. The anti-icing applications of superhydrophobic coatings are currently in the basic performance evaluation stage and are primarily focused on laboratory-based discussions and analyses. The anti-icing ideas are still focused on static droplet delayed icing and ice adhesion reduction and lack the study of dynamic droplet anti-icing and water droplet impact processes.

Since ordinary superhydrophobic coatings easily lose their superhydrophobic properties, researchers propose to prepare superhydrophobic coatings with high mechanical stability and self-repairing/self-healing functions. To further increase the effectiveness of superhydrophobic surfaces in de-icing, researchers suggest a technique that combines active de-icing with passive anti-icing to further increase the effectiveness of superhydrophobic surfaces in de-icing. This technique applies photothermal or electrothermal effects to superhydrophobic coatings and then illuminates or electrifies them to achieve a significant de-icing effect with superhydrophobic coating. However, many superhydrophobic coatings have not yet been applied on a large scale, and related performance tests have been confined to the laboratory. Therefore, performance testing of superhydrophobic coatings should focus on real-world environments. In the upcoming decades, the technique combining active de-icing and passive anti-icing will be spread throughout the anti-/de-icing industry.

## Figures and Tables

**Table 1:** Adhesion magnitude of superhydrophobic coatings with different epoxy resin contents [73].

FS%	CA (°) before Test	CA (°) after Test	Adhesion Evaluation
Without adhesive polymer (0%)			
1%	155	118.44	0B
1.5%	158.3	123.65	0B
2%	160	134.1	0B
2.5%	163.5	141.35	0B
Polymer (1%)			
1%	146.2	141.12	2B
1.5%	153.94	149.33	2B–3B
2%	154.81	152.07	2B–3B
2.5%	156.98	154.87	2B–5B
Polymer (2%)			
1%	128.65	127.22	4B
1.5%	140	137.14	4B
2%	152.17	150.92	4B–5B
2.5%	154.39	153.75	4B–5B
Polymer (3%)			
1%	119.82	119.0	5B
1.5%	138.34	138.0	5B
2%	154.1	154.1	5B
2.5%	154.61	154.61	5B

**Table 2:** The advantages and disadvantages of conventional anti-icing methods and superhydrophobic anti-icing methods.

Anti-Icing and De-Icing Technology	Advantage	Disadvantage
Mechanical de-icing and anti-icing technology	Effective de-icing and easy removal of thick ice	High machine cost, waste of manpower, easy to break the ring on the material surface
Hot melt de-Icing and anti-icing Technology	The clear de-icing effect, short de-icing time, the wide application range	High energy consumption, easy to start a fire
Chemical-based de-icing and anti-icing technology	Lower the melting point of ice and completely remove the ice layer	Pollute the environment and corrode material surfaces
Superhydrophobic de-icing and anti-icing technology	Resource-saving, low-cost, and anti-icing effect	Poor mechanical stability and easy failure at low temperatures

## Data Availability

The data supporting the findings of this study are available in the article.
